# Sea Otters Homogenize Mussel Beds and Reduce Habitat Provisioning in a Rocky Intertidal Ecosystem

**DOI:** 10.1371/journal.pone.0065435

**Published:** 2013-05-24

**Authors:** Gerald G. Singh, Russell W. Markel, Rebecca G. Martone, Anne K. Salomon, Christopher D. G. Harley, Kai M. A. Chan

**Affiliations:** 1 Institute for Resources, Environment & Sustainability, University of British Columbia, Vancouver, British Columbia, Canada; 2 School of Resource and Environmental Management, Simon Fraser University, Burnaby, British Columbia, Canada; 3 Department of Zoology and Biodiversity Research Centre, University of British Columbia, Vancouver, British Columbia, Canada; McGill University, Canada

## Abstract

Sea otters (*Enhydra lutris*) are keystone predators that consume a variety of benthic invertebrates, including the intertidal mussel, *Mytilus californianus*. By virtue of their competitive dominance, large size, and longevity, *M. californianus* are ecosystem engineers that form structurally complex beds that provide habitat for diverse invertebrate communities. We investigated whether otters affect mussel bed characteristics (i.e. mussel length distributions, mussel bed depth, and biomass) and associated community structure (i.e. biomass, alpha and beta diversity) by comparing four regions that varied in their histories of sea otter occupancy on the west coast of British Columbia and northern Washington. Mussel bed depth and average mussel lengths were 1.5 times lower in regions occupied by otters for >20 years than those occupied for <5 yrs. Diversity of mussel bed associated communities did not differ between regions; however, the total biomass of species associated with mussel beds was more than three-times higher where sea otters were absent. We examined alternative explanations for differences in mussel bed community structure, including among-region variation in oceanographic conditions and abundance of the predatory sea star *Pisaster ochraceus*. We cannot discount multiple drivers shaping mussel beds, but our findings indicate the sea otters are an important one. We conclude that, similar to their effects on subtidal benthic invertebrates, sea otters reduce the size distributions of intertidal mussels and, thereby, habitat available to support associated communities. Our study indicates that by reducing populations of habitat-providing intertidal mussels, sea otters may have substantial indirect effects on associated communities.

## Introduction

The capacity of top predators and ecosystem engineers to structure communities and affect ecological processes has been documented globally in a variety of systems, from mussel-dominated rocky intertidal shores [1] to subtidal rocky reefs [2], fish dominated rivers [3] and pelagic systems [4]; nematode dominated soil communities [5], and wolf dominated mountain forests [6]. While top predators are known to drive top-down processes [7], ecosystem engineers are recognized for their roles in creating or modifying habitat [8]. When the two interact, profound ecological changes can ensue, making the understanding of the effects of these species a central focus in ecology and conservation biology [7], [9], [10].

Sea otters (*Enhydra lutris*) are widely known for their strong indirect effects, via consumption of sea urchins, on shallow rocky reef and kelp forest ecosystems [2], [11], [12], [13], [14]. Otters, however, consume a wide variety of benthic invertebrates, including snails, crabs, clams, and sea stars [15], [16], [17]. In the absence of predation by otters, as a result of enhanced longevity, invertebrate prey frequently become “hyper-abundant” and attain much larger sizes [18], [19], [20]. Despite these well-known effects on shallow subtidal populations and communities, few studies have investigated how otters influence intertidal communities (but see [21], [22], [23]), and none to our knowledge on the structurally complex mussel beds that are characteristic of temperate rocky intertidal communities throughout the northeast Pacific.

The California mussel (*Mytilus californianus*) is a competitively dominant species, well known for its ability to exclude other sessile organisms that attach to primary substrate [24] and change diversity at local scales [1]. Mussels are also ecosystem engineers [25] that facilitate a variety of marine invertebrates by providing secondary substrata and structurally complex habitat across large spatial scales [26]. Diversity of mussel-associated species is related to mussel bed complexity [27] and positively correlated to mussel bed thickness, mussel biomass, and the amount of sediment trapped by mussel beds [25]. Otters, however, readily consume mussels, swallowing small mussels whole and breaking large mussels open with rocks [21], [28], and thereby have potentially widespread but poorly known negative indirect effects on mussel-associated communities.

Understanding the effects of sea otters on mussel beds may be confounded, however, by the effects of another keystone predator, the sea star, *Pisaster ochraceus*, (hereafter referred to as “*Pisaster*”), bottom-up and abiotic effects that regulate conditions for mussel recruitment, growth, and survival. *Pisaster* feed at the lower edge of mussel beds and can limit the lower extent of mussel bed distributions and, through size-selective foraging, increase heterogeneity and change species diversity of primary space occupiers within intertidal communities [1], [29]. However, *Pisaster* can also denude mussel beds and remove habitat for mussel-associated species, especially when coupled with a warming climate [30].

Mussel beds are also structured by oceanographic and other abiotic processes including water temperature, upwelling dynamics and wave exposure [31], [32], [33]. Mussel growth rates vary with water temperature and nutrient availability and, therefore, coastal upwelling dynamics [32]. Nutrient-rich upwelled waters support higher phytoplankton productivity, higher mussel growth rates, and larger mussels [32] and transport of mussel larvae onshore is correlated with higher recruitment rates and denser mussel beds [34].

In this study, we addressed the following questions. First, do areas with different histories of sea otter occupation have correspondingly different mussel bed structural characteristics? Second, if so, do mussel beds with different structural characteristics support different mussel-associated communities? We predicted that mussel length distributions would be reduced, mussel bed depths would be shallower, and mussel biomass lower in regions of longer otter occupancy time. Given the above, we predicted that mussel-associated communities should have higher biomass and species diversity in the prolonged absence of sea otters.

We investigated alternative hypotheses concerning the confounding effects of abiotic and bottom-up processes and the top-down effects of *Pisaster*. If oceanographic processes account for mussel bed characteristics, we expected that larger mussels would be found in regions where water temperature is generally higher, and that deeper mussel beds would correspond to areas with stronger upwelling. If *Pisaster* is largely the driver of mussel bed characteristics, we expected to find a higher proportion of smaller mussels, shallower mussel beds, and lower distributional limits of mussels to be higher on the shore where *Pisaster* are more abundant. There are multiple drivers of change in mussel beds, and the results of this study indicate that otters may be a significant but as-of-yet largely unexplored driver of mussel bed structure and communities.

## Methods

### Study system and experimental design

**Figure 1:**
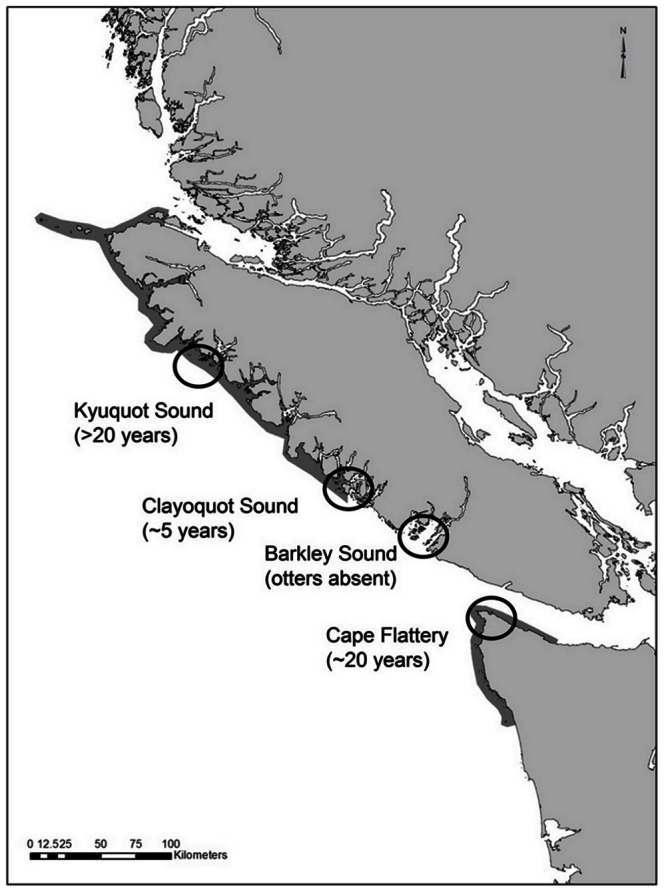
The regions included in this study. Shaded grey areas of the coast indicate the extent of otters at the time of sampling.

This study was conducted in four regions spanning the west coast of Vancouver Island, British Columbia and the northwest coast of Washington state (Figure 1) using a space-for-time substitution approach [35]. Among-region differences in sea otter reintroduction and range expansion has created a natural experiment in which regions differ in their recent history of sea otter occupancy and predation rates. These regions were Kyuquot Sound (otters present since before 1990, [13], [36]), Cape Flattery (otters present since 1990, [37]), Clayoquot Sound (otters present since about 2001, [36]), and Barkley Sound (otters not yet established, [38]). Within each region, we sampled three replicate sites. All sites were chosen based on similar observed conditions of wave exposure, slope, and species composition. Because otters were well established at both the northernmost and southernmost sites, we were able to avoid (to the extent possible) confounds associated with latitude. Collections permissions were granted by Fisheries and Oceans Canada, and permission to conduct field work on traditional lands were granted by the Makah Nation (Cape Flattery), the Huu-ay-aht First Nations (Barkley Sound), the Ahousaht First Nations (Clayoquot Sound), and the Kyuquot-Checleset First Nations (Kyuquot Sound).

### Mussel bed characteristics and associated community structure

All measurements of mussel bed distributional limits were measured relative to Canadian tidal datum; lower low water, large tide (LLWLT). At each site, ten 25×25 cm plots were randomly chosen at the middle (272–301 cm above LLWLT of vertical range) and lower (228–251 cm above LLWLT of vertical range) extent of mussel beds along a contour line. Average mussel size in each plot was determined by haphazardly choosing and measuring 15–50 mussels along the longest linear dimension. Fifty mussels were taken in regions where plots were removed from the mussel bed for quantification of species richness (Barkley Sound and Kyuquot Sound, see below) and 15–30 mussels were removed in regions where plots were not taken in their entirety (Clayoquot Sound and Cape Flattery). Haphazard sampling was implemented by removing mussels from the bed, mixing them in a bucket, and selecting the first X number of mussels. Mussel bed depth was determined by measuring the depth at which a knitting needle could be pushed into each bed, normal to the rock surface, until hitting bedrock.

In Kyuquot Sound and Barkley Sound, where sea otter occupancy differed the most (i.e. >20 years vs. otters absent), ten 25×25 cm plots were excavated from lower and mid mussel beds at three sites in each region (the same plots that mussel size were sampled from). Mussel biomass was measured by aggregating and weighing live mussels after having separated them from all mussel-associated species and sediment. Mussel-associated community biomass was measured by aggregating and weighing in the field all mussel bed species other than mussels. The mussel-associated community from each plot was then taken to the lab, fixed in formalin and preserved in a series of ethanol treatments [25], and later sorted and identified. A random 453.6 g subsample was taken from each mussel-associated community sample and sorted to the lowest taxonomic class possible (henceforth referred to as “morphospecies”). The weight of each morphospecies was recorded and the proportion of weight of each species group was multiplied by the total weight of the plot's mussel-associated species to calculate an estimate of the morphospecies for the plot. In the few samples where vertebrate fish were found, total lengths were estimated in the field while individual biomass was estimated based on published length-weight relationships for those species, or for morphologically similar species when necessary. For *Xiphister mucosus* we used the relationship for *Pholis gunnellus* [39], and for *Gobiesox maeandricus* we used the relationship for *Lepadogaster lepadogaster* [40].

### Regional oceanography

We used monthly sea surface temperature (SST) over 10 years and monthly upwelling index (UI) over 20 years (obtained from http://las.pfeg.noaa.gov) to quantify the variation in SST and UI among regions and the degree to which these oceanographic variables could explain differences in mussel bed structure. These data were used to build time series across regions based on yearly aggregated data. Mean upwelling and temperature indices were calculated to compare yearly averages among regions.

### Sampling Pisaster ochraceus

In the three northern regions we conducted surveys in the lower intertidal to quantify the density and size distributions of *Pisaster*. In each site (n = 3 per region) we randomly placed ten 1 m×1 m quadrats in the lower intertidal (along the lower extent of *M. californianus* beds and into the *S. sessile* zone). In each quadrat we counted and measured the maximum diameter of all *Pisaster*. We used length-weight regressions established with field-collected individuals to determine *Pisaster* biomass.

In all four regions, we measured the lower extent of mussel beds as an indication of the pressure of *Pisaster* foraging in mussel beds, as these seastars feed at the lower extent of beds while sea otters are not so spatially restricted. In Kyuquot Sound and Barkley Sound, these surveys were conducted at five sites while in Clayoquot Sound and Cape Flattery surveys were conducted at three sites. These sites (in Kyuquot and Barkley Sound) included the sites chosen for mussel bed community sampling. Five measures were taken haphazardly at every site. Sites in Washington were converted to Canadian chart datum (LLWLT) by comparing tidal projections at Makah Bay (in Washington) to Port Renfrew (across the Straight of Juan de Fuca on Vancouver Island) at Canadian tidal heights at 0 m over 2 years and finding the difference in tidal heights. This gave a correction factor (76 cm) to apply to the Washington mussel lower limits to estimate them at LLWLT. We also accounted for differences in tidal amplitude (Barkley Sound 582 cm, Clayoquot Sound 586 cm, Cape Flattery 602 cm, Kyuquot Sound 612 cm) on measures of lower extent by multiplying the lower extent above LLWLT by an index of the relative tidal amplitude (0–1, scaled to the maximum tidal amplitude in each region). This adjustment standardizes for the effect of emersion time [41], which differs at a fixed (absolute) shore level across a gradient of tidal amplitude, and thereby allowed us to isolate any effects of predation on the position of the lower limit of the mussel bed [42].

### Data analyses

All analyses (except where specified) were performed using R [43]. To test the effects of region on mussel bed characteristics, we used nested mixed effects models fit with Unequal Variance Restricted Maximum Likelihood (REML, [44]). We used models previously developed for regional comparisons using hierarchical sampling [45]. We treated Region as a fixed effect and Site as a random effect. The distributions of residual errors were graphically assessed for normality and homoscedasticity. Tukey post-hoc tests were used for multiple comparisons of mussel sizes and mussel bed depth. Because different sample sizes were used in different regions, we represented mussel sizes with probability density histograms, where the area under the curve integrates to one. We tested for differences in mussel size distributions using Kolmogorov-Smirnov tests.

To compare community composition between an otter present and otter absent region we used Nonmetric Multi-Dimentional Scaling (NMDS) and nested Analysis of Similarity (ANOSIM) tests with Bray-Curtis dissimilarities. We used a nested ANOSIM using sites as replicates because of the hierarchical design of the sampling, and because of the limited replication, only ten permutations were possible for analysis. As a consequence, significance levels are set at ά = 0.1 to protect against type II errors [46]. To supplement this analysis, we used hierarchical cluster analysis with Bray-Curtis dissimilarties. Complete linkages were used to prevent clusters being aggregated based on nearest elements being close together despite most elements in each cluster being distant from each other [47].

Species accumulation curves were modeled to estimate the total richness of the two extreme regions of otter occupancy time (i.e. otter >20 years vs. otters absent). Species accumulation curves estimate species richness by plotting the cumulative number of species discovered as a function of the number of individuals collected in the order they were observed. Species accumulation curves are recommended for estimating species richness with non-random spatial sampling [48]. We calculated diversity indices (Shannon-Weiner and beta diversity) to test for differences (using a nested mixed effect model) in biodiversity between regions differing in otter occupancy time. We calculated beta diversity as Whittaker's species turnover, β_W_  =  γ/α - 1, where γ is regional richness and α is plot richness. Beta diversity is a measure of community diversity among habitats.

We used Similarity Percentages (SIMPER) analyses to determine which species contributed most to differences in community structure between regions. Mussel bed biomass data were square-root transformed to minimize the impact of highly dominant morphospecies in this analysis. NMDS, ANOSIM, diversity indices, and SIMPER analyses were conducted using PRIMER 6. Species accumulation curves were analyzed with the ‘vegan’ package in R [49].

## Results

### Mussel bed characteristics

**Figure 2:**
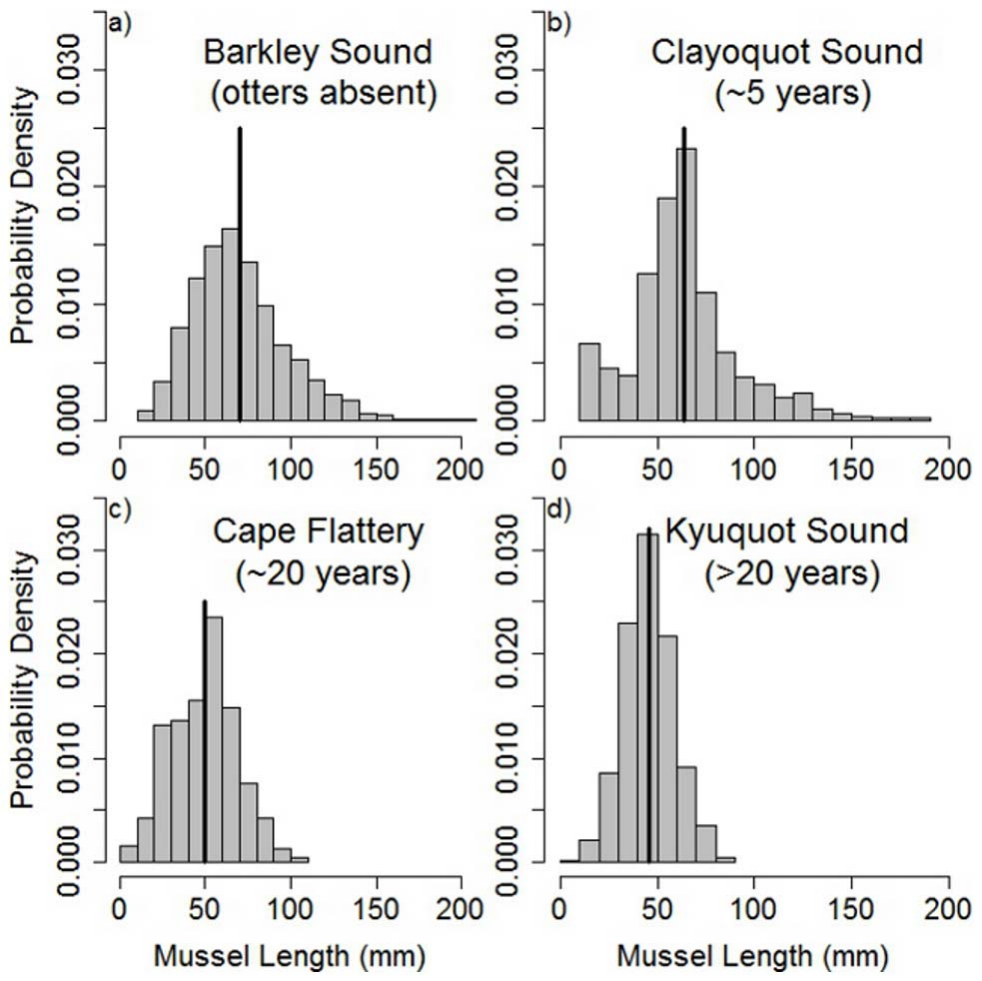
Size distributions of mussels in the four regions sampled. Mean sizes of mussels are indicated for each region by the vertical bar. Sample size for the different regions are 1368 (Barkley Sound), 519 (Clayoquot Sound), 450 (Cape Flattery), and 1018 (Kyuquot Sound).

We found smaller mussels and truncated length-frequency distributions associated with increasing sea otter occupancy time (Figure 2). Mussels from Barkley Sound (otters absent) and Clayoquot Sound (otters <5 years) were larger than mussels from Kyuquot Sound (otters >20 years) and Cape Flattery (otters ∼20 years) (*F*_3,9_ = 28.05, *p* = 0.0001). Kolmogorov-Smirnov tests indicated that distributions of mussel lengths differed between Barkley Sound (otters absent) and Clayoquot Sound (*p*<0.01), between Clayoquot Sound and Cape Flattery (*p*<0.01), and between Cape Flattery and Kyuquot Sound (*p*<0.01).

**Figure 3:**
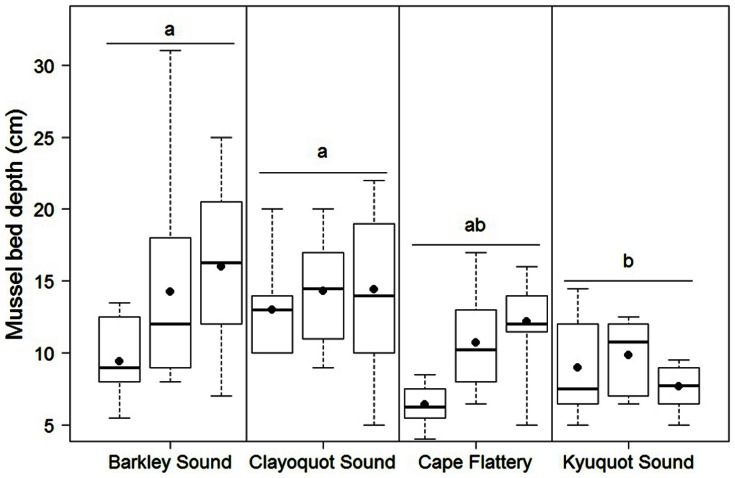
Depths of mussel beds among the regions sampled in this study. The three boxplots (horizontal bar: median, box: 25% and 75%, whiskers: minimum and maximum values) within each region represent three replicate sites within said region, and dots represent site means. Different lower-case letters indicate significantly different mussel bed depths among regions.

The depths of mussel beds also decreased with increasing history of sea otter occupancy (Figure 3). Mussel beds were deeper (*F*_3,8_ = 9.06, *p* = 0.006) in Barkley (absent) and Clayoquot (<5 years) Sounds than they were in Kyuquot Sound (>20 years). Variability in the depths in mussel beds also decreased with increasing sea otter occupancy (Figure 3).

**Figure 4:**
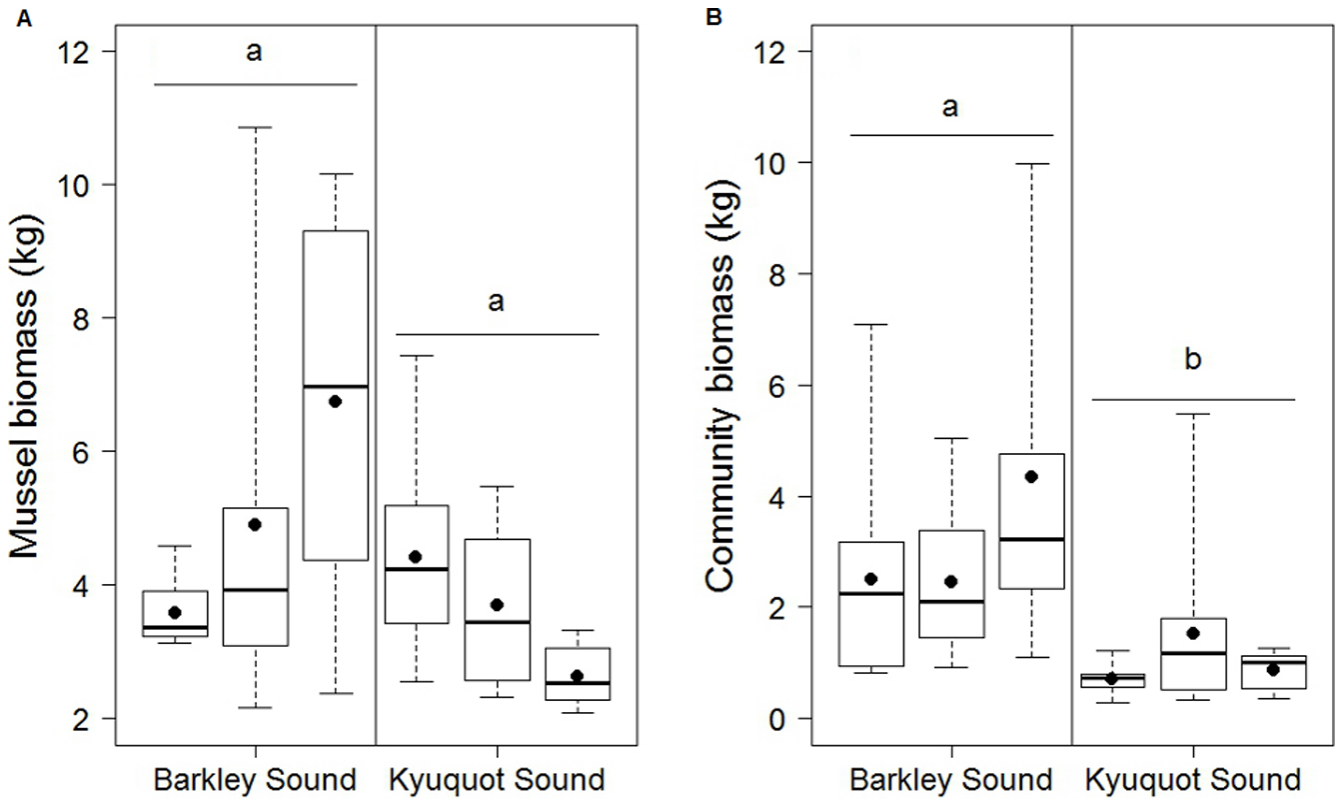
Biomass of mussels and mussel bed associated communities between regions with and without otters. (A) Biomass of mussels and (B) mussel bed associated community in a 25×25 cm plot, in Barkley Sound (otters absent) and Kyuquot Sound (otters present). The three boxplots (horizontal bar: median, box: 25% and 75%, whiskers: minimum and maximum values) within each region represent the three sites sampled in each region. Dots represent site means. Different lower-case letters indicate significantly different mussel bed depths among regions.

Correspondingly, the biomass of mussels in Barkley Sound (absent) tended to be higher than in Kyuquot Sound (Figure 4a), but not significantly (t = −1.43, df = 4, *P* = 0.23). The total biomass of the mussel-associated taxa (Figure 4b) was greater (t = −3.86, df = 4, P = 0.018), and the biomass of sediment and shell remains (data not shown) was greater (on average three times greater, t = −2.88, df = 4, *P* = 0.045) in Barkley Sound (absent) than in Kyuquot Sound (>20 years).

### Regional differences in oceanography

Although all four regions demonstrated similar annual patterns of upwelling, the two northern regions (Clayoquot and Kyuquot) varied similarly, while the two southern regions (Barkley and Cape Flattery) tended to mirror one another (Figure S1). Between 1990 and 2010, the mean index of coastal upwelling was highest in Cape Flattery (∼20 years), followed by Barkley Sound (absent), Clayoquot Sound (<5 years) and Kyuquot Sound (>20 years). SST demonstrated less consistent regional pairings than upwelling (Figure S2).

Mean SST from 1999 to 2009 was highest in Clayoquot Sound (<5 years), followed by Barkley Sound (absent), Kyuquot Sound (>20 years), and Cape Flattery (∼20 years). Kyuquot Sound was more similar to Clayoquot Sound and Barkley Sound in some years (e.g. 2005–2006) than Cape Flattery, and more like Cape Flattery in others (e.g. 2007–2008). Barkley Sound sometimes had the coldest sea surface temperatures (e.g. 2000–2002) and sometimes was one of the warmest regions (e.g. 2004–2009). Cape Flattery was sometimes a relatively cold region (e.g. 2004–2009) and sometimes a relatively warm region (e.g. 1999–2001). Clayoquot Sound was sometimes closest to Cape Flattery in temperature (e.g. 1999–2003) and sometimes more similar to Barkley Sound (e.g. 2004–2006). Immediately preceding sampling, Barkley Sound and Clayoquot Sound had similar SST, while Cape Flattery and Kyuquot Sound had similar SST (Figure S2).

### Regional differences in Pisaster abundance

Densities of *Pisaster ochraceus* (mean # m^−2^ ± SE) differed among regions (*F*_2,6_ = 8.60, *P* = 0.017), with densities highest in Barkley Sound (16.5±1.82), followed by Kyuquot Sound (10.70±1.18), and Clayoquot Sound (5.04±1.09). A Tukey post-hoc test identified Barkley Sound as having significantly higher densities than Clayoquot Sound (*P*<0.001), but not Kyuquot Sound (*p* = 0.057).

Biomass of *Pisaster* (mean g m^−2^ ± SE) did not differ (*F*_2,6_ = 2.47, *P* = 0.165) between Barkley Sound (0.54±0.15), Clayoquot (0.79±0.24), and Kyuquot Sound (0.11±0.03). Similarly, the tidal-amplitude corrected lower extent of mussel beds did not differ (*F*_3,12_ = 0.734, *P* = 0.55) between Barkley Sound (210.99±5.62 cm), Clayoquot Sound (216.25±7.80), Cape Flattery (214.93±12.66), or Kyuquot Sound (228.0±4.09).

### Mussel bed associated community structure

**Figure 5:**
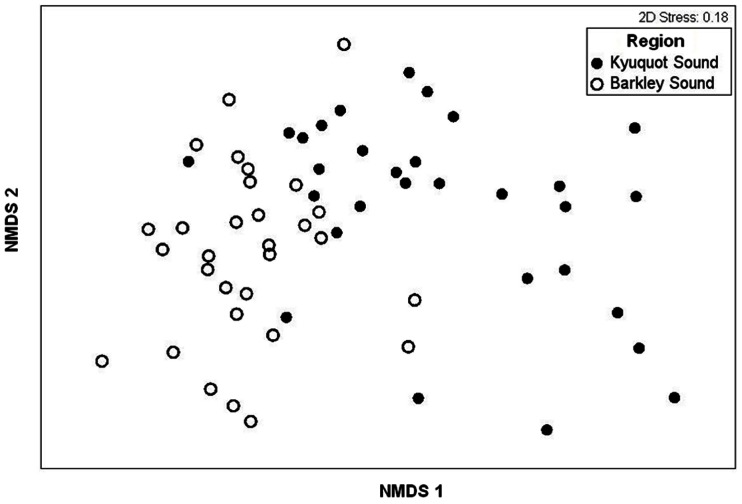
Community structure between regions with and without sea otters. NMDS plot showing separation of the two regions based on invertebrate community structure. Dots represent square-root transformed biomass data from 25×25 cm mussel quadrats. The axes (NMDS 1 and 2) are dimensionless but correspond to the greatest variance among the multivariate data points.

Mussel bed associated communities from Barkley (absent) and Kyuquot (>20 years) generally formed two NMDS clusters (Figure 5) that were distinct at the significance level chosen (nested ANOSIM, R = 0.63, *p*≈0.1). Among-site differences were smaller (nested ANOSIM, R = 0.19, p≈0.001) than among-region differences. Cluster analysis further indicated that individual plots clustered into two major groups; Barkley Sound (otters absent, 67% of the plots in the cluster) and Kyuquot Sound (otters present, 89% of the plots in the cluster) (Figure S3).

**Figure 6:**
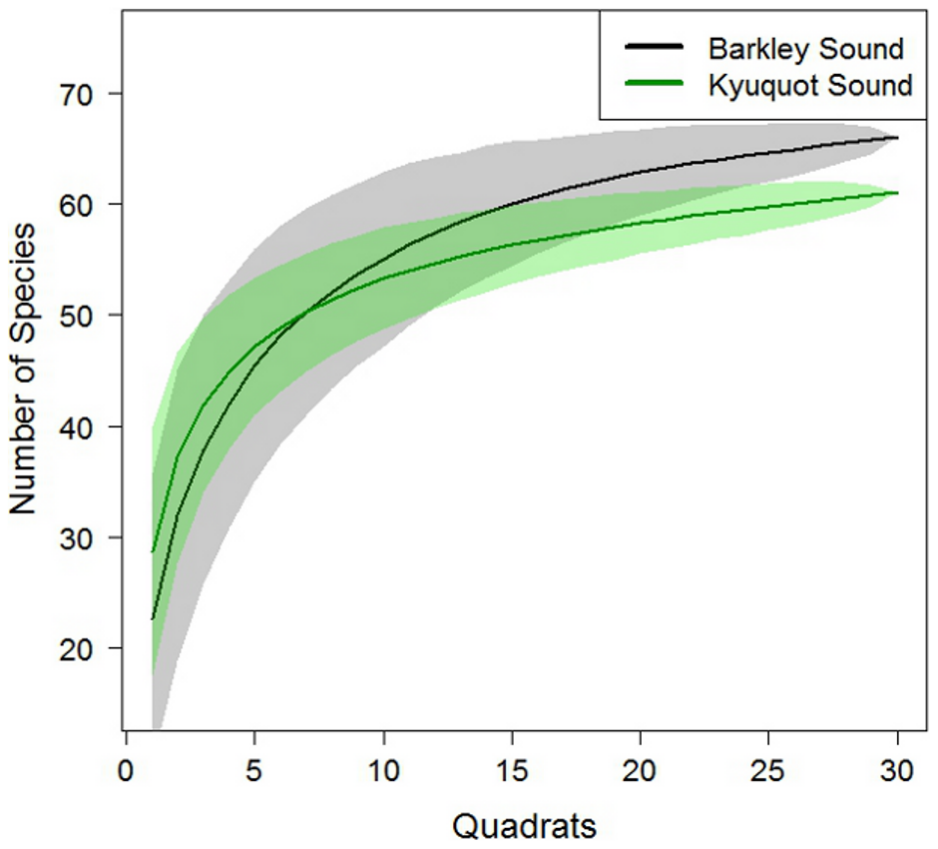
Estimates of species richness in regions with and without sea otters. Species accumulation curves for Barkley Sound and Kyuquot Sound. Average and bootstrapped 95% confidence intervals (shaded polygons) are based on 1000 permutations.

Our bootstrapped estimates of species richness from species accumulation curves indicated that mussel bed associated communities from Barkley (absent) have more species than those in Kyuquot (>20 years); 69.6 species vs. 64.1 species, respectively (Figure 6).

Based on Shannon diversity index values (mean ± SE), diversity did not differ (t = 0.17, df = 58, *p* = 0.86) between Barkley (1.14±0.07) and Kyuquot (1.16±0.11) Sounds. Values of beta diversity were not significantly different (t = −0.78, df = 3.9, *p* = 0.48) between Barkley (2.16±0.17) and Kyuquot (1.95±0.21) either.

The species accounting for the largest portions of the variation in mussel bed communities between Barkley (absent) and Kyuquot (>20 years) - summing to 75% of total variation - are shown in Table 1. Large barnacles, shore crabs, clams, cucumbers, isopods, peanut worms, and predatory snails make up this list. *Semibalanus cariosus*, *Pollicipes polymerus*, *Petrolisthes cinctipes*, and *Hiatella arcitca* together make up 53% of the dissimilarity between regions, and have between 1.7–3.6 times higher biomass in Barkley Sound than in Kyuquot Sound. Community biomass of mussel-associated species was approximately three-times higher in Barkley Sound than in Kyuquot Sound. All species (except *Nucella ostrina*) included in this table are included in the ten most abundant species found in each region. All species (except for *Cucumaria pseudocurata* and *Annelid worms*) had higher biomass in Barkley Sound.

**Table 1:** The species that contribute to the greatest dissimilarity in community biomass between regions.

Species	Functional group/Common name	Biomass (g) Kyuquot (>20 yrs)	Biomass (g) Barkley (absent)	Dissimilarity Index	Contribution (%) to Dissimilarity
*Semibalanus cariosus*	Barnacle	17.62	30.33	11.73	23.23
*Pollicipes polymerus*	Barnacle	5.41	12.77	6.96	13.78
*Petrolistes cinctipes*	Crab	2.45	8.93	4.83	9.56
*Hiatella arcitca*	Clam	1.99	7.36	3.7	7.32
*Cucumaria pseudocurata*	Sea cucumber	4.98	3.65	2.45	4.86
*Cirolana harfordi*	Isopod	3.2	5.09	2.17	4.3
*Nucella canaliculata*	Predatory snail	1.1	3.75	2.05	4.05
*Leukoma staminea*	Clam	1.18	2.66	1.72	3.4
*Phascolosom agassizii*	Peanut worm	1.6	2.72	1.71	3.38
*Nucella ostrina*	Predatory snail	0.73	1.6	0.97	1.93

## Discussion

### Effects of sea otters on mussel bed characteristics

Our results support the hypothesis that sea otter predation prevents mussels from attaining larger sizes and forming complex multi-layer beds. The structural characteristics of mussel beds measured in this study varied consistently with sea otter occupancy time. We found that mussels were largest where otters are absent, and smallest where otters have been present for long periods of time. Given that mussels are able to grow to large sizes (>150 mm), we argue that this phenomenon results from sea otters selectively removing large individuals while recruitment continues to supply small mussels [21], [51], [52]. In the absence of otters, many species – including urchins, abalone, and clams – are more abundant and attain larger sizes [18], [19], [20], [50]. Here we argue that intertidal mussels can be included in this list of invertebrate species regulated, in part, through sea otter predation. Some authors have not found mussel beds to change following sea otter reintroduction [53], though these authors acknowledge that otters may have removed large patches post-sampling.

Mussel bed depth was also correlated with otter occupancy time. Even within mussel beds, mussel bed depth can be quite variable due to abiotic stressors such as wave exposure [54], time since last disturbance, and the sizes and layering behaviour of mussels. Despite this potential variation, we found significant differences between regions, with deep beds where otters are absent and shallow beds where they are present. Crowding in mussel beds often leads to layering as the availability of primary substrate is reduced. The removal of mussels from beds and the underlying substrate by sea otters would thereby decrease bed depth.

Given that mussels are larger, and mussel beds are generally deeper where otters are absent, it is not surprising that we also found that the amount of sediment accumulated within mussel beds is also higher where otters are absent. Deeper, complex mussel beds can entrain sediment, and species diversity is positively associated with quantity of sediment in mussel beds [27]. Sediment is used as habitat and/or food source for polychaete worms, clams, and peanut worms [26], [55].

Despite mussels being much larger in the absence of sea otters, the biomass of mussels (per unit area) did not differ between regions; however, this may have resulted from small sample size (n = 3 sites) and particularly high among-site variability in Barkley Sound, where older mussel beds with larger mussels experience greater disturbance and recovery in patches [31]. Additionally, self-thinning is a process known to occur in mussel beds [56], and this process may have contributed to the lack of significant difference between regions found in this study. High densities of small mussels in Kyuquot Sound (>20 years) and lower densities of large mussels in Barkley Sound (absent) frequently resulted in similar estimates of mussel biomass. However, all explanations given here (low sample size, self-thinning) cannot be verified without further evidence.

We conclude that these multiple lines of evidence (smaller mean sizes, narrower size distributions, and shallower mussel bed depths) indicate that these regional-scale differences in mussel bed structural characteristics may plausibly be the result of varying sea otter occupancy times and corresponding predation rates.

### Alternative hypotheses: sea star predation and oceanographic conditions

Our regional comparisons of *Pisaster* indicate that this keystone predator is not likely responsible for inter-regional differences observed in our mussel bed parameters of interest. The size distribution of mussels in this study did not follow the expected pattern of mostly small mussels with few very large ones where *Pisaster* is abundant. Rather, the largest mussels and deepest mussel beds were sampled in the regions with highest abundances of *Pisaster*. The positive correlation between *Pisaster* abundance and large mussel sizes itself may be a result of otter absence, as otters are known to prey on both mussels and sea stars [15]. The lower boundary of mussel beds is regulated by *Pisaster* predation and by tidal exposure effects on productivity (recruitment and growth) so that the lower boundary falls at the shore level on which occurs a phase shift in the equilibrium between production and predation [42], [57], [58]. *Pisaster* typically forage on the lower edge of mussel beds, and can prevent mussel beds from extending into the lower intertidal zone [1], but we did not find differences in the lower extent of mussel beds corresponding to differences in *Pisaster* populations.

While differences in mussel beds were detected among regions varying in otter occupancy time, these differences did not in general correspond to patterns of key oceanographic variables or abundance of the keystone intertidal predator, *Pisaster*. Our regional comparisons of upwelling and SST indicate that these oceanographic variables do not vary consistently with regional patterns of mussel sizes, however we found a concordance of SST and mussel bed characteristics around the years that we sampled. Mussel growth is dependent on temperature and food availability [32], [59] and there can be an association between mussel recruitment and upwelling intensity [33]; thus these factors could lead to denser mussel beds with larger mussels. We do not have time series data on micro-habitat conditions (a scale at which mussels would respond), but at least we would expect to see mussel bed characteristics between regions with similar oceanographic properties to co-vary. We found this pattern in SST in the years immediately prior to sampling, which could indicate a role of oceanography in shaping our results. However, it can take upwards of five years for mussels to attain a length greater than 8–10 cm, and longer still to grow larger [29], so this period of covariance between mussel beds and oceanography may not be long enough to lead to the differences we see between regions.

We do not discount the importance of multiple drivers of change in mussel beds (including temperature, upwelling, and other predators), but argue and provide evidence that otters also play a key role, and in this case may be a plausible driver in structuring mussel beds.

### Biomass of mussel-associated communities

Mussels are competitive dominants in rocky intertidal communities, able to grow over and exclude other primary space occupiers [24]; however, their structural characteristics provide important secondary substrate and habitat for numerous sessile and mobile invertebrates. Larger mussels provide more surface area for epibionts to occupy, older mussels (often larger mussels) allow more time for epibiont colonization, sediment provides substrate for species and collects food, and deeper beds provide shelter from wave action, fluctuating temperatures, and desiccation stress [25]. We found that the total biomass of mussel-associated communities was three-times higher in the otter-absent region, where mussels are larger, beds are deeper, and there is more sediment. Large barnacles (*Semibalanus cariosus* and *Pollipes polymerus*) contributed over a third of the dissimilarity in community biomass between regions (Table 1). Both of these species are long lived (maximum age estimated at 15–20 years; [60], [61]) and are the dominant mussel-associated species. That a greater abundance of long-lived species is found in deeper beds with larger mussels suggests that mussels play a facilitative role and provide stable habitat for associated species for long periods of time. Our findings indicate that sea otters may have importance indirect effects on energy flows through rocky intertidal food webs, though further research is required to determine what, if any, effect sea otters might have on rocky shore productivity.

### Diversity of mussel-associated communities

Although higher micro-habitat diversity is generally associated with higher species diversity [62], in this study we did not find strong relationships between mussel bed structural characteristics and the diversity of associated mussel bed communities. This result contrasts with the findings of Kanter [27], [55], who found greater diversity in deeper beds with larger mussels. The different findings between these studies may be a consequence of a difference in the regional species pool and latitudinal gradients in diversity. Our work was conducted in British Columbia, Canada and Kanter [27], [55] conducted his work in California. The estimate of regional species richness for our study was just under 70 while in California over 100 species were recorded. A difference in methodology might also explain the contrasting findings, because our taxonomic resolution did not go to species in some cases (e.g. we considered polychaete worms as a single group). Finally, differences in diversity might be a response to global climate change since Kanter's work was conducted, as in 2006 a study [25] found reduced richness and no effect of mussel bed structure on diversity in the same area of California as Kanter, and attributed these differences to climate change.

Estimates of community richness and diversity show remarkable similarity between regions (based on bootstrapped richness estimates, Shannon diversity indices and beta-diversity). This suggests that regional pools of species are similar between otter present and otter absent areas, and that higher variability in mussel bed structure does not result in higher species accumulation. This result was surprising, given that we expected to see greater variation among plots in Barkley Sound, where we found greater variation in mussel sizes and mussel bed depth and therefore presumably greater variation in available niche space among mussel beds.

However, to our knowledge, our study is the first to examine the identity of species in mussel beds that contribute most to dissimilarity between communities of different mussel bed structure, and therefore provides mechanistic insight into this finding. We found that the species that contribute most to the dissimilarity of mussel-associated community diversity between regions are also most dominant within each region. These species are barnacles that attach and grow on mussel shells (∼2 times greater biomass where otters absent), clams that usually burrow (∼3 times greater biomass where otters absent), and free-living decapods, echinoderms, isopods, gastropods, and sipunculid worms (∼2 times greater biomass where otters absent) that could be subject to wave dynamics in the absence of appropriately sized refuge habitats as are found in the beds of large mussels [63].

## Conclusions

Our study builds on and integrates concepts of facilitation by ecosystem engineers and strong per capita interaction strength of keystone species. It also suggests an addition to the list of the driving forces of intertidal communities (e.g. SST, upwelling, exposure) and expands what is known about the community ecology of sea otters by specifically exploring how sea otters influence intertidal communities. By investigating how a large, mobile keystone predator interacts with a mid-trophic level ecosystem engineer, this study demonstrates how negative interactions (predation) can hinder positive interactions (facilitation through habitat formation). The mussel bed attributes investigated in this study decrease in variability across a gradient of sea otter influence, and biomass of associated species is lower where otters are present; this phenomenon can be attributed to otters homogenizing mussel bed structure and decreasing the function of mussels as habitat providers. However, this phenomenon can also be seen from another perspective – that large mussels and deep beds with high community biomass, similar to large urchins, clams, and other invertebrates [18], [19], [20], [50], are a by-product of sea otter extirpation. As sea otter populations continue to grow and re-colonize historic ranges shallower mussel beds with smaller mussels and lower community biomass may be a return to population and community dynamics that precede human-induced sea otter extirpation.

## Supporting Information

Figure S1 **Time series of upwelling among the four regions in the study.** Annual upwelling anomaly (difference from a global mean) for the four regions in the study, from 1990 to 2010. Upwelling patterns do not vary consistently with regional patterns of mussel bed characteristics measured in this study.
(TIF)

Figure S2 **Time series of Sea Surface Temperature (SST) among the four regions in the study.** Annual sea surface temperature anomaly (difference from a global mean) for the four regions in this study, from 1999 to 2009. SST patterns do not vary consistently with regional patterns of mussel bed characteristics measured in this study.
(TIF)

Figure S3 **Mussel bed communities in each quadrat group according to region (otters present or absent).** A hierarchical cluster analysis on the mussel bed associated communities in both regions. Of the two distinct clusters, one is predominantly made of plots from Barkley Sound (otters absent), while the other is predominantly made up of plots from Kyuquot Sound (otters present).
(TIF)
