# Supplementary figures and images for: Sea Otters Homogenize Mussel Beds and Reduce Habitat Provisioning in a Rocky Intertidal Ecosystem
Source: PLoS One. 2013 May 24;8(5):e65435. doi: 10.1371/journal.pone.0065435 (PMC3663835; doi:10.1371/journal.pone.0065435)

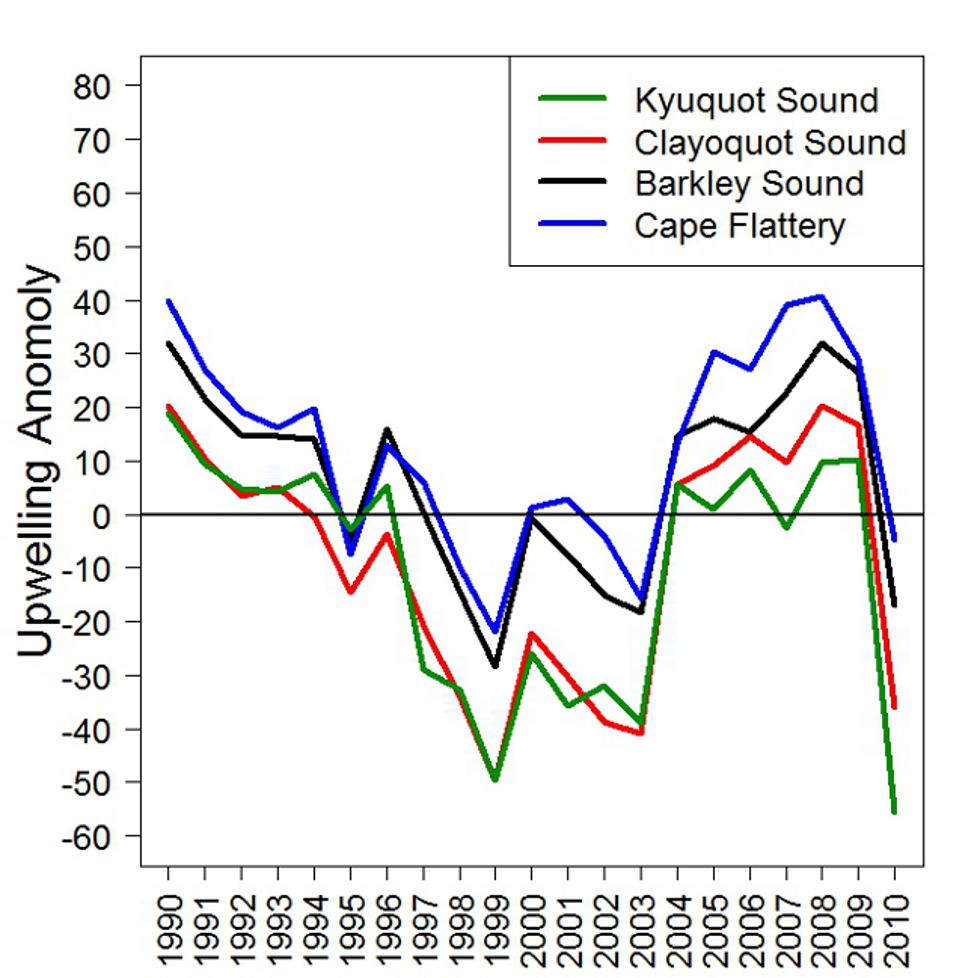

Supplement: Figure S1 — Time series of upwelling among the four regions in the study. Annual upwelling anomaly (difference from a global mean) for the four regions in the study, from 1990 to 2010. Upwelling patterns do not vary consistently with regional patterns of mussel bed characteristics measured in this study. (TIF) [file pone.0065435.s001.tif]

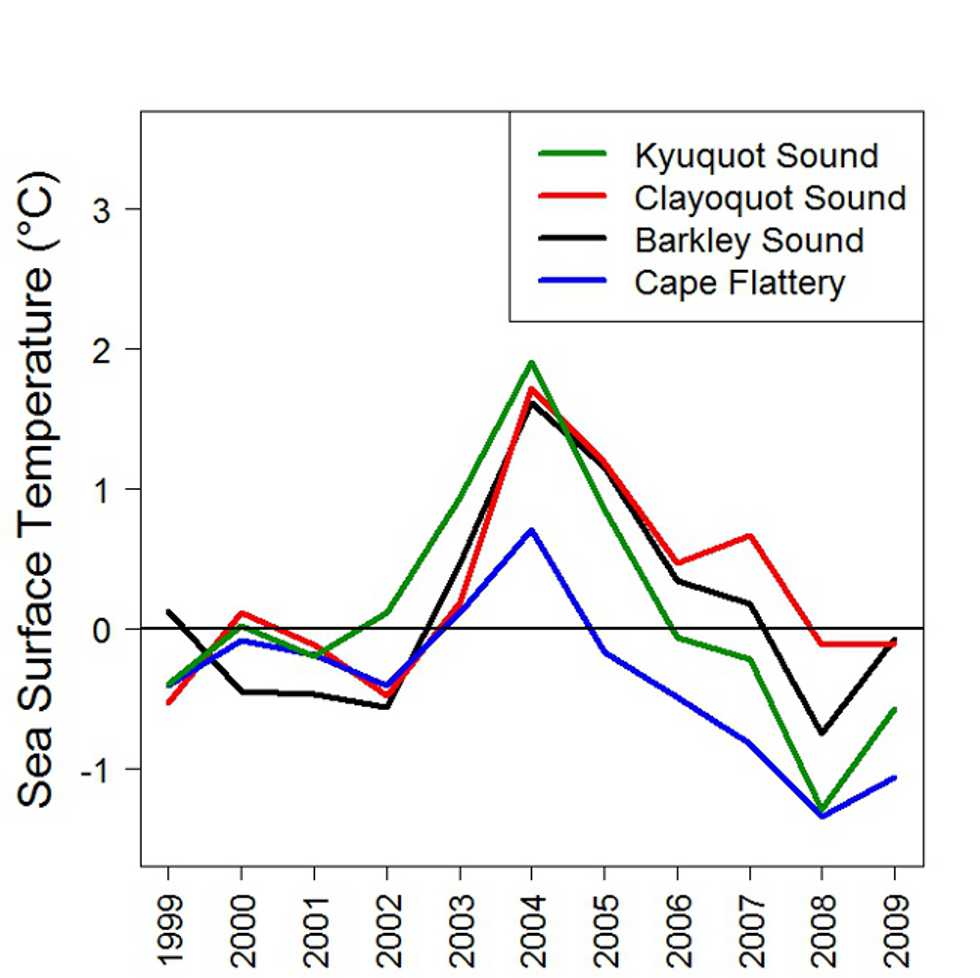

Supplement: Figure S2 — Time series of Sea Surface Temperature (SST) among the four regions in the study. Annual sea surface temperature anomaly (difference from a global mean) for the four regions in this study, from 1999 to 2009. SST patterns do not vary consistently with regional patterns of mussel bed characteristics measured in this study. (TIF) [file pone.0065435.s002.tif]
